# Supplementary material for: Heparin-based hydrogel scaffolding alters the transcriptomic profile and increases the chemoresistance of MDA-MB-231 triple-negative breast cancer cells
Source: Biomater Sci. 2020 Feb 13;8(10):2786–96. doi: 10.1039/c9bm01481k (PMC7497406; doi:10.1039/c9bm01481k)
Supplement: Supplementary file 2 [file BM-008-C9BM01481K-s002.zip › Supplementary File 4/HP-BhydrogelvControl/Pathways/my_analysis.Gsea.1545201079907/HALLMARK_KRAS_SIGNALING_DN.html]

Details for gene set HALLMARK\_KRAS\_SIGNALING\_DN[GSEA]

|  || Dataset | expr.class.cls#HYD\_versus\_CONTROL.class.cls#HYD\_versus\_CONTROL\_repos |
| Phenotype | class.cls#HYD\_versus\_CONTROL\_repos |
| Upregulated in class | HYD |
| GeneSet | HALLMARK\_KRAS\_SIGNALING\_DN |
| Enrichment Score (ES) | 0.3832876 |
| Normalized Enrichment Score (NES) | 1.4091535 |
| Nominal p-value | 0.038926173 |
| FDR q-value | 0.12797786 |
| FWER p-Value | 0.623 |
Table: GSEA Results Summary

  

Fig 1: Enrichment plot: HALLMARK\_KRAS\_SIGNALING\_DN      
 Profile of the Running ES Score & Positions of GeneSet Members on the Rank Ordered List

  

| PROBE | DESCRIPTION (from dataset) | GENE SYMBOL | GENE\_TITLE | RANK IN GENE LIST | RANK METRIC SCORE | RUNNING ES | CORE ENRICHMENT || 1 | SPTBN2 | na |  |  | 140 | 3.123 | 0.0423 | Yes |
| 2 | DTNB | na |  |  | 412 | 2.458 | 0.0671 | Yes |
| 3 | CHRNG | na |  |  | 656 | 2.157 | 0.0887 | Yes |
| 4 | DLK2 | na |  |  | 668 | 2.145 | 0.1222 | Yes |
| 5 | UPK3B | na |  |  | 759 | 2.067 | 0.1503 | Yes |
| 6 | ATP6V1B1 | na |  |  | 1205 | 1.738 | 0.1546 | Yes |
| 7 | CHST2 | na |  |  | 1302 | 1.689 | 0.1764 | Yes |
| 8 | IDUA | na |  |  | 1707 | 1.488 | 0.1789 | Yes |
| 9 | SHOX2 | na |  |  | 1871 | 1.418 | 0.1929 | Yes |
| 10 | TGM1 | na |  |  | 2054 | 1.339 | 0.2046 | Yes |
| 11 | KCNN1 | na |  |  | 2328 | 1.231 | 0.2099 | Yes |
| 12 | NR6A1 | na |  |  | 2330 | 1.231 | 0.2294 | Yes |
| 13 | MAST3 | na |  |  | 2434 | 1.197 | 0.2430 | Yes |
| 14 | CLSTN3 | na |  |  | 2487 | 1.177 | 0.2590 | Yes |
| 15 | SYNPO | na |  |  | 2788 | 1.089 | 0.2606 | Yes |
| 16 | NRIP2 | na |  |  | 2841 | 1.074 | 0.2749 | Yes |
| 17 | MTHFR | na |  |  | 3061 | 1.022 | 0.2797 | Yes |
| 18 | P2RX6 | na |  |  | 3489 | 0.921 | 0.2720 | Yes |
| 19 | CPEB3 | na |  |  | 3727 | 0.864 | 0.2733 | Yes |
| 20 | HSD11B2 | na |  |  | 3749 | 0.858 | 0.2858 | Yes |
| 21 | MYO15A | na |  |  | 3834 | 0.844 | 0.2948 | Yes |
| 22 | KCND1 | na |  |  | 3976 | 0.817 | 0.3004 | Yes |
| 23 | TFCP2L1 | na |  |  | 4036 | 0.805 | 0.3101 | Yes |
| 24 | HTR1D | na |  |  | 4082 | 0.793 | 0.3204 | Yes |
| 25 | GP1BA | na |  |  | 4183 | 0.773 | 0.3274 | Yes |
| 26 | STAG3 | na |  |  | 4228 | 0.765 | 0.3373 | Yes |
| 27 | GTF3C5 | na |  |  | 4371 | 0.738 | 0.3415 | Yes |
| 28 | CELSR2 | na |  |  | 4443 | 0.725 | 0.3493 | Yes |
| 29 | EGF | na |  |  | 4542 | 0.703 | 0.3554 | Yes |
| 30 | EPHA5 | na |  |  | 4702 | 0.675 | 0.3578 | Yes |
| 31 | EDN1 | na |  |  | 4838 | 0.651 | 0.3611 | Yes |
| 32 | SERPINB2 | na |  |  | 4996 | 0.627 | 0.3628 | Yes |
| 33 | MX1 | na |  |  | 5402 | 0.570 | 0.3507 | Yes |
| 34 | MSH5 | na |  |  | 5655 | 0.531 | 0.3459 | Yes |
| 35 | LFNG | na |  |  | 5702 | 0.525 | 0.3518 | Yes |
| 36 | FGFR3 | na |  |  | 5871 | 0.504 | 0.3510 | Yes |
| 37 | RIBC2 | na |  |  | 5872 | 0.504 | 0.3590 | Yes |
| 38 | H2AFY2 | na |  |  | 5914 | 0.498 | 0.3648 | Yes |
| 39 | CAMK1D | na |  |  | 5991 | 0.487 | 0.3686 | Yes |
| 40 | FGGY | na |  |  | 6002 | 0.485 | 0.3758 | Yes |
| 41 | SPHK2 | na |  |  | 6006 | 0.484 | 0.3833 | Yes |
| 42 | ABCG4 | na |  |  | 6690 | 0.394 | 0.3538 | No |
| 43 | THRB | na |  |  | 6885 | 0.372 | 0.3496 | No |
| 44 | MAGIX | na |  |  | 7099 | 0.348 | 0.3440 | No |
| 45 | MFSD6 | na |  |  | 7341 | 0.321 | 0.3364 | No |
| 46 | BTG2 | na |  |  | 7702 | 0.284 | 0.3221 | No |
| 47 | PRODH | na |  |  | 7745 | 0.282 | 0.3244 | No |
| 48 | TCF7L1 | na |  |  | 8165 | 0.238 | 0.3063 | No |
| 49 | BARD1 | na |  |  | 8323 | 0.217 | 0.3015 | No |
| 50 | ENTPD7 | na |  |  | 8606 | 0.191 | 0.2898 | No |
| 51 | CCDC106 | na |  |  | 8623 | 0.190 | 0.2919 | No |
| 52 | GPRC5C | na |  |  | 9069 | 0.145 | 0.2710 | No |
| 53 | SGK1 | na |  |  | 9100 | 0.141 | 0.2716 | No |
| 54 | NR4A2 | na |  |  | 9329 | 0.112 | 0.2615 | No |
| 55 | PTPRJ | na |  |  | 9349 | 0.110 | 0.2622 | No |
| 56 | CACNA1F | na |  |  | 10030 | 0.036 | 0.2272 | No |
| 57 | SLC25A23 | na |  |  | 10168 | 0.019 | 0.2204 | No |
| 58 | TAS2R4 | na |  |  | 10223 | 0.015 | 0.2178 | No |
| 59 | IFI44L | na |  |  | 10300 | 0.015 | 0.2140 | No |
| 60 | SLC6A14 | na |  |  | 10335 | 0.014 | 0.2125 | No |
| 61 | CCNA1 | na |  |  | 10576 | -0.014 | 0.2001 | No |
| 62 | EFHD1 | na |  |  | 10897 | -0.053 | 0.1842 | No |
| 63 | KRT15 | na |  |  | 11062 | -0.073 | 0.1768 | No |
| 64 | C5 | na |  |  | 11091 | -0.078 | 0.1766 | No |
| 65 | SLC29A3 | na |  |  | 12020 | -0.176 | 0.1308 | No |
| 66 | PDK2 | na |  |  | 12032 | -0.178 | 0.1331 | No |
| 67 | GPR3 | na |  |  | 12105 | -0.187 | 0.1323 | No |
| 68 | GAMT | na |  |  | 12257 | -0.208 | 0.1277 | No |
| 69 | ITGB1BP2 | na |  |  | 12418 | -0.222 | 0.1228 | No |
| 70 | GPR19 | na |  |  | 12454 | -0.222 | 0.1245 | No |
| 71 | YPEL1 | na |  |  | 12708 | -0.244 | 0.1152 | No |
| 72 | SNN | na |  |  | 12897 | -0.271 | 0.1096 | No |
| 73 | COPZ2 | na |  |  | 13658 | -0.379 | 0.0759 | No |
| 74 | TGFB2 | na |  |  | 14509 | -0.492 | 0.0392 | No |
| 75 | CDKAL1 | na |  |  | 14624 | -0.511 | 0.0414 | No |
| 76 | RSAD2 | na |  |  | 14852 | -0.544 | 0.0381 | No |
| 77 | PDE6B | na |  |  | 14919 | -0.553 | 0.0435 | No |
| 78 | PLAG1 | na |  |  | 15658 | -0.678 | 0.0156 | No |
| 79 | SKIL | na |  |  | 16151 | -0.777 | 0.0022 | No |
| 80 | EDN2 | na |  |  | 16304 | -0.811 | 0.0071 | No |
| 81 | SLC16A7 | na |  |  | 16366 | -0.825 | 0.0170 | No |
| 82 | PTGFR | na |  |  | 16553 | -0.866 | 0.0211 | No |
| 83 | CYP39A1 | na |  |  | 17115 | -1.019 | 0.0079 | No |
| 84 | TFF2 | na |  |  | 17346 | -1.100 | 0.0133 | No |
| 85 | BMPR1B | na |  |  | 17678 | -1.234 | 0.0156 | No |
| 86 | ASB7 | na |  |  | 18557 | -1.749 | -0.0026 | No |
| 87 | FAM46C | na |  |  | 18953 | -2.269 | 0.0128 | No |
Table: GSEA details [plain text format]

  

Fig 2: HALLMARK\_KRAS\_SIGNALING\_DN      
 Blue-Pink O' Gram in the Space of the Analyzed GeneSet

  

Fig 3: HALLMARK\_KRAS\_SIGNALING\_DN: Random ES distribution      
 Gene set null distribution of ES for **HALLMARK\_KRAS\_SIGNALING\_DN**
